# Cost Analysis of a Novel Enzymatic Debriding Agent for Management of Burn Wounds

**DOI:** 10.1155/2017/9567498

**Published:** 2017-02-15

**Authors:** Giuseppe Giudice, Angela Filoni, Giulio Maggio, Domenico Bonamonte, Michelangelo Vestita

**Affiliations:** ^1^Section of Plastic and Reconstructive Surgery, Department of Emergency and Organ Transplantation, University of Bari, 11 Piazza Giulio Cesare, 70124 Bari, Italy; ^2^Section of Dermatology, Department of Biomedical Science and Human Oncology, University of Bari, 11 Piazza Giulio Cesare, 70124 Bari, Italy

## Abstract

*Introduction*. Given its efficacy and safety, NexoBrid™ (NXB) has become part of our therapeutic options in burns treatment with satisfactory results. However, no cost analysis comparing NXB to the standard of care (SOC) has been carried out as of today.* Aim*. To assess the cost of treatment with NXB and compare it to the SOC cost.* Methods*. 20 patients with 14–22% of TBSA with an intermediate-deep thermal burn related injury were retrospectively and consecutively included. 10 of these patients were treated with the SOC, while the other 10 with NXB. The cost analysis was performed in accordance with the weighted average Italian Health Ministry DRGs and with Conferenza Stato/Regioni 2003 and the study by Tan et al. For each cost, 95% confidence intervals have been evaluated.* Results*. Considering the 10 patients treated with NXB, the overall savings (total net saving) amounted to 53300 euros. The confidence interval analysis confirmed the savings.* Discussion*. As shown by our preliminary results, significant savings are obtained with the use of NXB. The limit of our study is that it is based on Italian health care costs and assesses a relative small cohort of patients. Further studies on larger multinational cohorts are warranted.

## 1. Introduction

Burn debridement with eschar removal, later followed by autograft surgery, is defined as the standard of care (SOC) in the management of burn patients [1]. Surgical debridement (escharectomy) may be performed early or delayed (as is the case at our burn center), as some authors believe that an accurate assessment of burn depth cannot be reached initially [1, 2]. Escharectomy is performed through tangential excision of layers of necrotic tissue until the bleeding healthy tissue is reached [2]; it is effective but also traumatic. Furthermore, it is not selective, and it determines loss of both viable tissue and blood and, of course, requires dedicated resources such as specialized personnel and operating facilities.

NexoBrid (NXB) [3] is a debriding enzyme mixed with an inert carrier gel forming a debriding gel dressing. NXB consists of a lyophilized, partially purified proteolytic protein mixture with increased specific enzymatic activity derived from bromelain raw material extracted from pineapple plant stems.

Several animal and human studies [3–11] have shown that NXB is a safe, effective, and selective method for early eschar removal, reducing the need for surgery while achieving long-term outcomes that are comparable to those of SOC, therefore being a valid alternative to conventional surgical SOC management for intermediate and intermediate-deep burns. In particular, such randomized controlled trials have demonstrated a significantly lower percentage of wounds needing surgical debridement, lesser time to complete eschar removal, and lower percentage of autograft surgery and blood loss in the NXB-treated group of patients.

NXB has been designated as an orphan drug in 2002 by the FDA and EMA (UE/3/02/107), and this designation has been confirmed in 2012 by the EMA EMA/COMP/631996/2012 recommendation.

In the present study, we aimed to analyze the effective cost of treatment of patients with intermediate-deep burns treated with NXB and compare it to the cost of treatment with SOC.

## 2. Materials and Methods

This cost analysis study was conducted at the Burn Unit of the University Hospital of Bari, Italy, retrospectively including 20 patients, admitted between January 2015 and June 2016, with intermediate or intermediate-deep thermal burns involving a total body surface area (TBSA) between 14% and 22%.

All patients were aged >18 years. Ten patients were treated with SOC, while the remaining 10 patients were treated with NXB.

In particular, the 10 patients in the NXB group with the abovementioned burn features were retrospectively recruited, choosing individuals consecutively treated from October 2015, the time point at which NXB became available at our institution, to June 2016. The 10 patients in the SOC group with the same burn features were also retrospectively recruited, enrolling subjects consecutively treated in the 9 months immediately before, that is, from January 2015 to September 2015.

SOC was defined as surgical escharectomy (first surgical step) and autograft surgery (second surgical step). In the 10 patients treated with NXB, the first surgical step was not performed. NXB dosage was based on the treated area and product characteristics. In particular, as per indication, 2 g of NXB powder (cost per unit 437.67 euros) dissolved in 20 g of gel is applied to a burn area of 100 cm^2^, which is about 1% of TBSA. In our study, we used the 5 g package (cost per unit 839.27 euros), which is to be applied to approximately 2.5% of TBSA.

NXB was applied in every case in an ambulatory or bedside setting 30 min after minor patient intravenous sedation and analgesia (1 mg midazolam, 5–10 mg morphine, and 1 g paracetamol). One physician and a nurse were involved in the process. After 4 h, the product was removed, and saline soaks were left in place for 4–10 h before proceeding with standard dressing.

The cost analysis was performed in accordance with the weighted average DRGs of the Italian Health Ministry [12], as well as values drawn from Conferenza Stato/Regioni 2003 [13] and Tan et al. [14]. In particular, the costs of hospitalization in the burn center intensive care unit (ICU) and subintensive care unit (SICU) concerned the following four cost components: diagnostics (medical imaging and laboratory services), consumables (drugs, fluids, and disposables), hotel and nutrition, and labor (specialists, nurses, and consulted specialists such as medical specialists, pharmacists, physiotherapists, laboratory technicians, and nutrition specialists) [12–14]. Real medical costs were used for units of concentrated blood cells and were calculated by multiplying the volumes of health care use with the corresponding unit prices. We evaluated the distribution of costs using STATA MP14 Sktest and because data were normally distributed, for each cost, 95% confidence intervals were evaluated.

All costs applied to the financial year 2012. Overheads and the specific costs of burn dressings were explicitly excluded from the cost analysis.

## 3. Results

Table 1 summarizes the anagraphical, clinical, and volume of care data of the 20 included patients, while Table 2 demonstrates the assessment of budget expenses and the cost comparison between the NXB and SOC groups, as well as the confidence interval analysis for each cost item in the two groups. Supplementary Table 1 in Supplementary Material available online at https://doi.org/10.1155/2017/9567498 shows the raw data used for analysis.

From Tables 1 and 2, we have the following:

- Patients in the NXB group stayed on an average 6 days in the ICU and 25 days in the SICU, whereas patients in the SOC group stayed 8 days on an average in the ICU and 29 days in the SICU. Considering an ICU cost per day of 1325 euros and an SICU cost per day of 475 euros, such reduction in terms of hospitalization determined a saving of 37075 euros in the NXB group.
- Patients in the NXB group did not undergo escharectomy, whereas all patients in the SOC group underwent escharectomy. Considering a cost per unit of 1675 euros and considering that three patients in the SOC group underwent two different escharectomy surgeries, a total saving of 21775 euros was determined in the NXB group.
- Six patients in the NXB group underwent autograft surgery, whereas all patients in the SOC group underwent autograft surgery. Considering a cost per unit of 12414 euros, a total saving of 49656 euros was determined in the NXB group.
- Five patients in the NXB group required transfusion with 1 unit of blood (RBC) each, whereas all patients in the SOC group required transfusion (three patients with 1 unit, six patients with 2 units, and one patient with 3 units). Considering a cost per unit of 208 euros, a total saving of 2704 euros was determined in the NXB group.
- The total net saving amounted to 53300 euros in the NXB group. This was derived from 111210 of saved resources (ICU∖SICU hospitalization, escharectomy, autograft surgery, and blood transfusion) (total gross saving) minus total NXB cost of 57910 euros for the assessed 10 patients.
- The 95% confidence interval analysis of costs demonstrated an interval of 34915.85–42423.7 for total costs in the SOC group and an interval of 20657.68–34677.14 for total costs in the NXB group.

## 4. Discussion

Management of burn patients is notoriously expensive in terms of patient hospitalization time, surgical procedures, transfusion, dressings, and other accessory therapeutic measures, and dedicated ICU personnel and related costs [15–17].

The clinical efficacy and safety of NXB have been demonstrated by a recent randomized controlled trial [3]. In fact, with enzymatic debridement, less surgery is required for both excising burns and sacrificing donor sites to obtain skin grafts to cover the excised wound beds; therefore, a reduction in hospitalization times can be attained. These characteristics also result in improved patient quality of life. Based on the above data, NXB has become a part of our therapeutic options in the treatment of burns with satisfactory results, as assessed by both physicians and patients.

However, to the best of our knowledge, this is the first assessment of budget impact investigating whether NXB use might be financially convenient besides being efficacious.

Our cost analysis was designed based on DRGs; these are weighted estimates of the costs sustained by the healthcare and correspond to the reimbursement received by the burn unit for each patient. In Italy, a DRG for a hospitalized burn patient who is not treated with any surgery amounts to a specific sum per day (*x*), while another patient who stays the same days but undergoes surgery has a higher DRG (*x* + *n*). We measured the economic impact of single procedures based on the differences in total DRG occurring among differently treated patients. Real medical costs were used for blood, since these are not considered in DRG estimates, and therefore eluded the analysis. Other escaping costs included dressing costs, and these have not been considered for a matter of simplification.

As shown by our results, noticeable savings, amounting to 53300 euros for 10 patients in our observation, were obtained with the use of NXB. In particular, the parameters that considerably differentiated the SOC and NXB groups were the ICU∖SICU length of hospitalization and the need for escharectomy and autograft surgeries. In our observation, the average saving per patient was 5330 euros. The confidence interval analysis confirmed the savings obtained in the NXB group when compared to those in the SOC group.

Of note, the Italian weighted DRGs do not permit differentiation of costs based on wound extension when considering escharectomy and autograft surgeries, as the DRG coding is the same for these procedures, regardless of the extension of the burn wounds to be excised or covered with a graft, respectively. This is certainly a limit of our study since we know that the use of NXB also reduces the percentage of wounds that will need an autograft surgery; however, with the Italian DRG system, a small autograft would cost as much as a larger one because of being coded by the same DRG. Nonetheless, it seems correct to presume that the need for less extensive autograft surgeries indirectly and positively influences other financial parameters such as the need for blood transfusions and, especially, the length of hospitalization.

Importantly, as per the data sheet, NXB should be applied to a maximum of 15% of TBSA as a safety measure since no data are yet available regarding its safety profile when used on a greater TBSA. However, there is an increasing trend to treat larger burn areas with a single NXB application, with appropriate patient monitoring, as of now, with successful results and an unchanged safety profile. We, among other authors, have collected encouraging data (unpublished) on the efficacy and safety of NXB when used in up to 22% of TBSA over the last year and a half.

In conclusion, our results are preliminary and reflect the Italian national health care costs and are possibly limited by the retrospective study design and Italian DRG coding limitations; they need to be confirmed by further observations of larger cohorts of patients, possibly in multinational studies. Regardless, at present, our results indicate a significant economic advantage associated with the use of NXB over the traditional SOC management of intermediate or intermediate-deep burns.

## Supplementary Material

Raw data of the two studied groups of burn patients (treated with either Nexobrid or standard of care) used for direct cost assessment as well as for confidence interval analysis.

## Figures and Tables

**Table 1:** Patients groups characteristics and volume of care data.

	SOC	NXB
Number of patients	10	10

Patients (male)	6	6

Average age (range)	49 (19–64)	48 (18–69)

Etiology	6 scalds 3 flame 1 contact burn∖flame	5 scalds 5 flame

Average % TBSA burns (range)	17 (14–21)	17 (15–22)

Average stay (days) (ICU + SICU) (range)	37 (29–42)	31 (26–38)

Average ICU stay (days) (range)	8 (5–12)	6 (4–9)
95% CI volume of care	6.1–9.2	5.1–7.5

Average SICU stay (days) (range)	29 (24–34)	25 (21–29)
95% CI volume of care	26.7–30.9	23.2–26.6

Escharectomy (unit × number of patients)	1 × 7 patients	0
	2 × 3 patients	
95% CI volume of care	0.95–1.64	0-0

Autograft (unit × number of patients)	1 × 10 patients	1 × 6 patients
95% CI volume of care	1-1	0.23–0.96

Blood Transfusion (unit × number of patients)	1 × 6 patients	1 × 5 patients
	2 × 2 patients	
	3 × 1 patients	
95% CI volume of care	0.7–1.9	0.12–0.9

NXB units consumed per patient		4 patients 6 NXB unit
		4 patients 7 NXB unit
		1 patients 8 NXB unit
		1 patients 9 NXB unit

NXB: Nexobrid; SOC: standard of care; ICU: intensive care unit; SICU: subintensive care unit; TBSA: total body surface area; CI: confidence interval.

**Table 2:** Cost analysis and relative and total savings. SOC versus NXB.

	SOC	NXB	NXB SAVING	95% CI costs SOC		95% CI costs NXB	
ICU stay cost (euros) (each 1325 euros)	102025	83475	18550	8082.5	12190	6757.5	9937.5
SICU stay cost (euros) (each 475 euros)	136800	118275	18525	12682.5	14677.5	11020	12635
Escharectomy cost (euros) (each 1675 euros)	21775	0	21775	1591.25	2747	0	0
Autograft cost (euros) (each 12414 euros)	124140	74484	49656	12414	12414	2855.22	11917.44
Blood transfusion (euros) (each 208 euros)	3744	1040	2704	145.6	395.2	24.96	187.2
Gross NXB saving			111210				

NXB cost (euros) (each 839.27 euros)		57910					

*Totals*	*388484*	*335184*	*53300*	*34915.85*	*42423.7*	*20657.68*	*34677.14*

NXB: Nexobrid; SOC: standard of care; CI: confidence interval.
